# Usability Evaluation of an Online, Tailored Self-Management Intervention for Chronic Obstructive Pulmonary Disease Patients Incorporating Behavior Change Techniques

**DOI:** 10.2196/resprot.2246

**Published:** 2013-01-16

**Authors:** Viola Voncken-Brewster, Albine Moser, Trudy van der Weijden, Zsolt Nagykaldi, Hein de Vries, Huibert Tange

**Affiliations:** ^1^CAPHRIDepartment of General PracticeMaastricht University Medical CenterMaastrichtNetherlands; ^2^Centre of Research on Autonomy and Participation of Persons with a Chronic IllnessFaculty of Health and CareZuyd University of Applied SciencesHeerlenNetherlands; ^3^University of Oklahoma Health Sciences CenterDepartment of Family and Preventive MedicineOklahoma City, OKUnited States; ^4^CAPHRIDepartment of Health PromotionMaastricht University Medical CenterMaastrichtNetherlands

**Keywords:** usability testing, Internet intervention, computer tailoring, chronic obstructive pulmonary disease, self-management

## Abstract

**Background:**

An eHealth intervention using computer tailored technology including several behavior change techniques was developed to support the self-management of chronic obstructive pulmonary disease patients.

**Objective:**

The goal of this study was to evaluate and improve the usability of the eHealth intervention.

**Methods:**

We conducted a usability evaluation with 8 chronic obstructive pulmonary disease patients, with a mixed methods design. We improved the usability through iterative cycles of evaluation and adaptation. Participants were asked to think aloud during the evaluation sessions. Participants then completed a semi-structured interview. The sessions were observed and recorded. Descriptive statistics and content analysis were used to uncover usability issues.

**Results:**

Areas for improvement were layout, navigation, and content. Most issues could be solved within 3 iterations of improvement. Overall, participants found the program easy to use. The length of the program urged us to further analyze the appreciation of behavior change techniques. Some were perceived as helpful and easy to use, while others evoked frustration.

**Conclusions:**

The usability study identified several issues for improvement, confirming the need for usability evaluation during the development of eHealth interventions. The uncovered strengths and limitations of behavior change techniques may lead to optimization of eHealth interventions, but further insight is needed.

## Introduction

Chronic obstructive pulmonary disease (COPD) is one of the major causes of morbidity and mortality worldwide [1]. COPD patients suffer a progressive deterioration of respiratory function along with significant systemic consequences [2]. Although the airflow limitation is not fully reversible, hospital admissions can be reduced and health-related quality of life can be improved by adequate patient self-management [3,4]. Self-management interventions should focus on behavior modification, such as smoking cessation, physical activity, and medication adherence, in order to attain improvement in health status [5]. Many home-based disease management programs have been developed to improve the health of chronically ill patients [6] including COPD patients [7]. These programs make use of eHealth.

EHealth interventions have become quite popular in number and reach. They are accessible over the Internet or mobile technologies and can offer information to support health-related behavior change to large population segments at any time with decreased personnel demands [8]. Especially the usage of computer-tailored technology for patient education and changing lifestyles is becoming increasingly popular in eHealth programs. Computer-tailored technology has been shown to effectively support behavior modifications [9,10]. For example, smoking cessation studies by Strecher et al [11] and Te Poel et al [12] showed higher continued abstinence rates in the group that received a Web-based tailored smoking cessation intervention compared to the control group. Computer tailoring principles can also be applied for changing multiple behaviors [13,14]. By tailoring feedback messages to a person’s responses, messages become more personalized and matched to key theoretical determinants of the behavior and characteristics of the person [15,16]. For example, a smoking relapse prevention program of Elfeddali et al [17] used name, gender, and motivational characteristics, such as perceived pros and cons of not smoking, levels of self-efficacy, and perceived stress. Personalization and adaptation of computer-tailored messages result in increased attention, appreciation, and processing of information [14,16].

Despite the growing popularity of eHealth interventions, it is very common for users who experience difficulties with the program to discontinue program use or drop out of a study before completion [18,19]. A critical factor for the uptake and retention of consumers for these programs is a high quality user-centered design [19]. To make a program efficient, effective, and satisfying to use, a usability study on the program can be conducted [20,21]. Usability studies enable developers to discover problems with the program and to explore end users’ experiences. In many practical usability engineering situations where it is not necessary to collect quantitative data for benchmark purposes, it is possible to gain sufficient insight with a small test group [22]. Iterative cycles of evaluation and adaptation can be followed to obtain this information to improve the prototype and increase its user-friendliness [20,23].

In the MasterYourBreath project (AdemDeBaas in Dutch), a computer-tailored program is accommodated to support self-managed behavior change of COPD patients. This paper reports findings from one of the first phases of this project—the evaluation of the usability of the program. A pragmatic approach was followed, with 3 iterations of detection and resolution of usability problems.

## Methods

### Design

This was an exploratory study with a mixed methods design. The study contained a usability evaluation with an iterative design to assess and improve the user-friendliness of the program. In addition, research was carried out to examine end users opinions on the behavior-change techniques (BCTs) integrated in the program.

### Recruitment

We recruited 8 Dutch speaking COPD patients from the Maastricht region, the most Southern part of the Netherlands. All participants were capable of using a computer. To create a heterogeneous sample of COPD patients, half the patients were recruited through their family doctor and the other half through flyers in the Maastricht University Medical Center.

### Ethical Considerations

This study was in accordance with all applicable regulations and was approved by the Medical Ethical Committee of Maastricht University Medical Center. All participants received an information letter about the study and participated in an informed consent procedure with a researcher. All participants received a gift voucher after the evaluation session.

### Prototype Description

The prototype described in this paper was a further development of existing computer-tailored programs for behavior change, originally developed for public health research. The Internet application Tailorbuilder (OverNite Software Europe, Sittard, NL) based on Perl and a MySQL5 database served as a container for domain-specific knowledge such as routing procedures, tailoring rules, and feedback messages.

The kernel of the program is a reasoning engine, which is based on the I-Change Model for behavior change [24,25]. It incorporates 8 BCTs in a predefined order (Table 1, [26,27]). Researchers can enter domain-specific knowledge about smoking cessation, physical activity, and other desired behaviors. This knowledge will be captured into specific questions, rules, and advices. Via a Web interface, patients and other users can access the program to seek advice. They can choose between different modules, including a general assessment module for health risk appraisal and 3 modules targeted to change specific behaviors. These behaviors are smoking cessation, medication adherence, and physical activity.

So far, the program has only been used in the general population. The modules for smoking cessation and physical activity were based on earlier projects [14,25] and were adapted for the COPD target group. The medication module was developed specifically for the MasterYourBreath project.

**Table 1:** BCTs defined by Michie et al [27].

BCTs used in this prototype	Definition
1. Provide information on consequences of behavior in general	Information about the relationship between the behavior and its possible or likely consequences in the general case, usually based on epidemiological data, and not personalized for the individual (contrast with technique 2).
2. Provide information on consequences of the behavior to the individual	Information about the benefits and costs of action or inaction to the individual or tailored to a relevant group based on that individual’s characteristic (ie, demographics, clinical, behavioral, or psychological information). This can include any costs or benefits and not necessarily those related to health (eg, feelings).
3. Provide information about others’ approval	Involves information about what other people think about the target person’s behavior. It clarifies whether others will like, approve, or disapprove of what the person is doing or will do.
4. Goal setting (behavior)	The person is encouraged to make a behavioral resolution (eg, do more exercise next week). This is directed towards encouraging people to decide to change or maintain change.
5. Barrier identification/problem solving	This presumes having formed an initial plan to change behavior. The person is prompted to think about potential barriers and identify the ways of overcoming them. Barriers may include competing goals in specified situations. This may be described as problem solving. If it is problem solving in relation to the performance of a behavior, then it counts as an instance of this technique. Examples of barriers may include behavioral, cognitive, emotional, environmental, social, and/or physical barriers.
6. Provide feedback on performance	This involves providing the participant with data about their own recorded behavior or commenting on a person’s behavioral performance or a discrepancy between one’s own performance in relation to others’.
7. Plan social support/social change	Involves prompting the person to plan how to elicit social support from other people to help him/her achieve their target behavior/outcome. This will include support during interventions (eg, setting up a buddy system or other forms of support and following the intervention including support provided by the individuals delivering the intervention, partner, friends, and family).
8. Prompt identification as a role model/position advocate	Involves focusing on how the person may be an example to others and affect their behavior (eg, being a good example to children). Also includes providing opportunities for participants to persuade others of the importance of adopting or changing the behavior (eg, giving a talk or running a peer-led session).

### Prototype Walkthrough

A typical scenario for using the program is as follows. After logging in to the program for the first time, participants filled out the assessment module, measuring smoking behavior, medication adherence, and physical activity. The assessment module consisted of demographical questions, questions to assess smoking behavior, the Fagerström Test for Nicotine Dependence [28], the Medication Adherence Report Scale (MARS-5) [29] to assess medication adherence, the Short Questionnaire to Assess Health-Enhancing Physical Activity (SQUASH) [30] to assess physical activity, and questions assessing the stages of change [31] and intention to change these 3 behaviors on a Likert scale. This questionnaire elicited health risk appraisal feedback, which contained information on the users’ lifestyle.

When participants logged in the second time, they were invited to choose from one of the behavior modules. The module started with the assessment of the motivational beliefs toward a particular behavior. Responses then generated tailored feedback. Consequently, the program assessed social influence on the behavior and provided feedback. Plans for behavior change were then assessed and tailored to the feedback in an action planning step. Finally, the program attempted to improve self-efficacy by identifying barriers participants experienced when changing or maintaining the behavior and by assessing plans that participants made to overcome these barriers. This yielded feedback on the identified barriers and approaches to overcome them. See Table 2 for an overview of the program including the BCTs used per intervention component.

**Table 2:** BCTs used in the program.

Users actions	Intervention components	BCTs integrated in the intervention components^a^
Users fill out the assessment module and receive health risk appraisal feedback	Questionnaire assessing demographics, intention for behavior change, stages of change, and a health risk assessment. Health risk appraisal feedback was based on the outcomes of the assessment.	1, 6
Users receive feedback in one of the modules (smoking cessation, physical activity, or medication adherence)	Motivational beliefs	1, 2
	Social influence	3, 7, 8
	Action planning	4
	Self-efficacy	5

^a^ These steps correspond to those shown in Table 1.

### Procedure and Data Collection

All evaluation sessions took place in a laboratory setting at Maastricht University Medical Center, except one, where a participant was visited at home due to his medical condition. The test scenario for the users was to log on, follow the instructions presented in the program, and complete the program. A test script for the moderator was written out in detail. Analysis guidelines were written out in advance. Prior to starting the usability study, the procedure was pretested on one individual, who was not participating in the usability study and not diagnosed with COPD, to ensure that all aspects of the usability evaluation would function adequately.

The study was performed in successive series of individual tests. For each series we asked 5 test subjects, or less if saturation was reached. We planned for 3 series, with the possibility to proceed should new usability problems arise continually. All series were conducted by the same moderator and observer, and both were not involved in the development of the program. The moderator guided the participants through the test but did not intervene or disrupt the thinking process. She would only provide help if participant explicitly requested help to proceed with the tests. Both the moderator and the observer noted the problems participants encountered.

**Figure 1:**
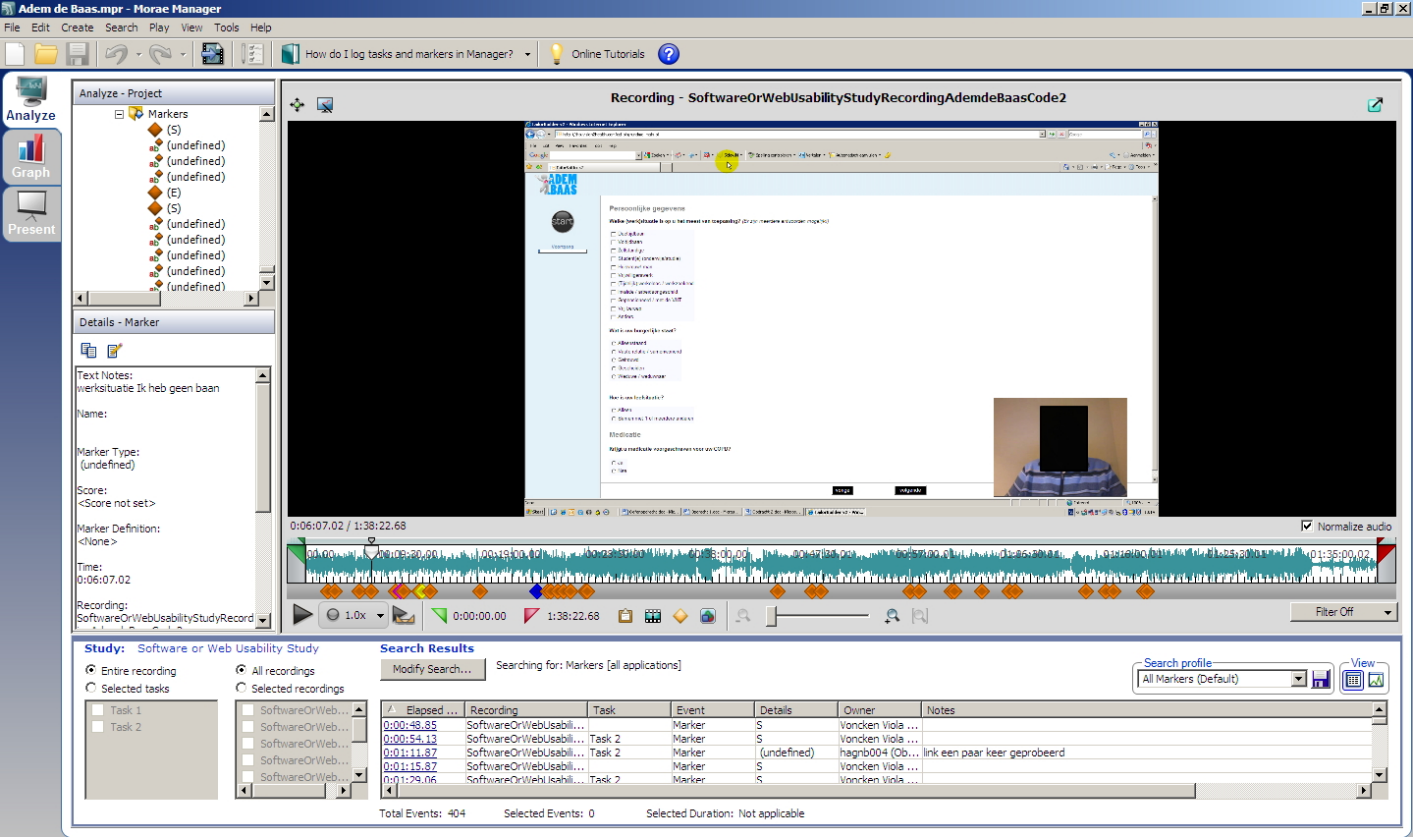
Screenshot of a recording.

Participants were asked to perform 2 tasks, which were the same as those that would be performed by future program users. The first task was to go to the website, log on, and complete the assessment module to receive health risk appraisal feedback. The second task was to complete one of the modules aimed at changing a specific behavior. Within each task, the users had no freedom of navigation, which assured that every participant encountered the same elements of the program. The think aloud method was used to assess participants’ reasoning and the source of their problems while using the computer program [32]. Participants were asked to verbalize their thoughts while performing the tasks. We emphasized that the intention was to evaluate the program and not the participants’ behavior, in order to encourage participants to talk freely. Because thinking aloud is an unusual task, participants were given one chance to practice this task. Morae video-analytic software (TechSmith Corporation, Okemos, MI, USA) was used to capture screen display, mouse clicks and participants’ verbal comments along with nonverbal reactions using a webcam. This allowed the moderator and observer to review the sessions to identify problems that were missed during the sessions. Figure 1 shows a screenshot of a recording of the pretest.

In the last step of each session, participants were interviewed about their experiences with the tasks and their prior computer experience. At the end of the interview, the users were asked to rate the program on a scale from 0 to 10, with 0 being very bad and 10 being excellent. The observer took field notes and composed a descriptive summary from each interview.

### Analysis

Descriptive statistics (ie, median and range) for task completion rate, completion time, program rating, and demographical characteristics of participants were computed. To uncover usability issues, the think aloud data, keystrokes, and mouse clicks were reviewed by both the observer and the moderator. They independently placed markers in the video recordings at times that participants encountered problems during task performance or commented on the content of messages. These markers, along with descriptive summaries, field notes, and the interviews, were used to identify the problems participants experienced working with the prototype. Quantitative criteria for usability problems were completion time, number of help questions, and number of errors. For the qualitative data, a content analysis approach was used [33]. Observer and moderator together grouped these problems into 3 categories (content, layout, and navigation) and identified the major problems from the list of problems. Major problems were system errors, problems that repeatedly occurred, problems that caused user irritation, and problems that were recalled by the user in the interviews. All other marked problems were labeled as minor problems. When no new major problems were identified by a participant, the results were used for program refinement. This process was repeated in the following rounds, until saturation was reached. The observer and moderator met frequently to discuss findings, interpretations, and data synthesis.

### Program Improvement

After each evaluation round, the detected problems of major importance were further classified into 2 groups. These groups were problems that could be solved with simple solutions implemented by the research team quickly before the next round of usability evaluation, and problems that required complex solutions. The solutions to the more complex problems had to be implemented by the vendor of the system, therefore these items were put on a wait list for a future upgrade.

## Results

### Participant Characteristics

The tests were conducted in 3 series. In the first round, 4 participants were tested, 2 in the second round, and 2 in the third round. Of the participants, 4 were recruited by their family doctor and 4 from the hospital. The ages of the 8 participants ranged from 51-70 years, (median 59.5). There were 5 male and 3 female participants. The education level of the participants varied—5 had a high level of education, 2 had an intermediate level, and 1 a low level of education. Internet experience ranged from 5-20 years, while current computer use varied from 3-21 hours per week. None of the participants were familiar with the prototype that was evaluated. The severity of disease ranged from mild COPD to very severe COPD, needing oxygen therapy.

### Usability Issues

The completion time of both tasks together varied from 42-120 minutes with a median of 67 minutes (71 minutes in round 1, 82 minutes in round 2, 59 minutes in round 3). Filling out the assessment module and receiving the health risk appraisal feedback (task one) took less time (15-39 minutes, median 29 minutes) than completing one of the modules aimed at changing a specific behavior (22-84 minutes, median 40 minutes). Completion rate was 100% in all cases. The number of help questions per participant varied from 0-2 (median 1) and the number of errors from 0-6 (median 1). Participants in the third round had the least usability problems, with only 1 help question (font size) and 1 navigation error (scrolling).

The participants’ rating of the program ranged from 6-9 on a 10-point scale (median 7.75). They were generally satisfied with the layout of the program. All participants were able to navigate through the program with minimal interference of the observer. During the interviews participants commented that the program was overall easy to navigate and the content was comprehensible. Nonetheless, suggestions for improvement were made. Analysis revealed specific problems in 3 domains: layout, navigation, and content. These problems and the changes we made after each round of testing are explained below and the major problems are summarized in Table 3. In the third round only some new minor problems arose as major problems reached saturation.

#### Layout

Most participants in the first round overlooked the information regarding the option of increasing the font size. As a solution, we improved the visibility of this instruction by repositioning it to the top left hand corner of the page, adding a title and increasing the font size of the instruction. All participants in the next round of testing noticed it.

Another problem concerned filling out a question matrix. A participant clicked in the wrong check box, which led to incorrect answers. This problem was solved by leaving a blank line after every 3 lines of questions with answer options.

A minor problem was uncovered in the third round of testing and related to the utilization of the unfolding questions. An unfolding question is a question that appears only when a certain answer is given to the previous question. The position of these questions confused a participant, because the unfolding question appeared right after the given answer instead of after the former question.

#### Navigation

Every participant completed both tasks without any navigation problems in the first round. In the second round of testing, participants noticed the instruction to change the font size, but this introduced 2 new navigation problems. Participants pressed the wrong keys in trying to increase the font size by pressing the key combination “Ctrl +”. Thereafter, we improved the instructions. Despite this change, a participant still struggled with increasing the font size in the last round of testing. Another navigation problem was linked to the need to scroll to view all of the information after increasing the font size. Three participants who increased the font size forgot to scroll down to see the rest of the page.

#### Content

We detected 4 major usability problems concerning the content of the questions and feedback messages. The first content problem manifested in the physical activity questionnaire (SQUASH) that was part of the health risk appraisal. Participants who were retired or unemployed were irritated by the 4 work/school related questions. One participant commented that “one would almost feel discriminated against”*.* All participants in the first round of testing used these questions to fill out the time they spent walking and bicycling at their leisure, being unaware of the fact that they would be asked about these activities later. As a consequence, they reported the same activities twice, which resulted in an overestimation of the amount of minutes spent walking and bicycling. We solved this problem by adding an option “I do not have a job/go to school” and changing the sequence of the questions (see Table 3 for changes across rounds). During the last round of testing, participants completed this part correctly.

The second problem concerned the perceived similarity of 2 questions—the question that assessed the stages of change and the question that measured the intention to change on a Likert scale. These 2 questions were very similar and all participants in the first round of testing perceived this as unnecessary and expressed frustration. We initially solved this by adding an explanation to the second question that these questions might look similar, but have different response options. In the second round participants showed no frustration, but still a participant commented that asking this question twice was unnecessary. We decided to eliminate the second question.

The third problem concerned the length of the program. As a matter of fact, each behavior module urges the user to pass through all BCTs, which takes time. One participant recommended shortening the feedback messages. Also, participants often did not take the time to read all feedback messages and commented that it was a lot to read.

The last problem was brought to our attention by a participant who suffered from severe COPD. He stated that the feedback was not appropriate to his situation, due to the severity of his condition. “If this is for people like me, there should be adjustments for functional limitations. Here they talk mainly about the possibilities, about people who are mobile etc., but the people who cannot get out of the house, for those adjustments should be made.” He also expressed that the term “physical activity norm”, which was used in the feedback, sounded too negative. He reflected that achieving the norm highly depends on a person’s possibilities to achieve the norm. We removed the term physical activity norm, but still gave the recommendation to be physically active for 30 minutes a day.

In addition to the above modifications, some minor changes were made. A participant suggested that an extra question about breathing problems during physical activity was missing. This question was added. A clarifying example for a question about medication intake was removed, since this led to confusion and incorrect responses. Also, some changes to word choice were made in response to comments. For example, one participant found the terminology “COPD patients” rather offending. She reasoned that a person is more than a patient. “I find the word COPD patient or cancer or lung patient a nasty slogan... I am not the disease, I have it.” We followed this recommendation and replaced “COPD patient” by “people with COPD”.

### Post-Hoc Analysis: Evaluation of the BCTs

One of the problems identified by the participants was that the program length was too long. As the length of the program was hindered by the requirement that all participants must pass through all the BCTs, we reanalyzed the think aloud data using content analyses to assess the users’ opinions about each BCT.

Participants agreed with the information on consequences of behavior in general (BCT 1) and talked about how this was applicable to their own lives. One participant thought that it was more useful to younger people, who are not yet familiar with the disease.

Opinions concerning BCT 2, information on the consequences of behavior to the individual, were mixed. One participant claimed to know all of the information presented in this section already, while another participant stated that the information may stimulate behavior change.

The opinions about the 3 BCTs focusing on social influence (BCT 3, 7, and 8) were mixed—comments were mainly negative for BCT 3 and 7, while some participants thought that BCT 8 gave good suggestions (ie, joining a sports club or finding a buddy to exercise with). Information about others’ approval (BCT 3) was bypassed by some participants. They indicated not to be concerned about what other people think or do. Prompting identification as a role model/position advocate (BCT 7) was not appreciated as some particiants thought that behaviors that require change should only be identified by themselves, not others. A participant considered this awkward and patronizing. “I find that hurtful... everybody has their own motives, you should not talk to people about that. I almost find that patronizing.” Another participant was afraid that his friends would not appreciate it if he talked to them about behavior change in a persuasive manner. However, this participant understood that he could also just set the right example instead and talk about his health achievements without trying to persuade others.

A variety of views were expressed towards BCT 4, goal setting (behavior). One participant had difficulties understanding the questions about plans they made for behavior change. Another participant thought that it was useful to make plans, while two other participants found planning useless. One argued that he was physically active without the need to think of a plan and felt that this part of the program was not applicable to him.

Five participants had difficulties identifying barriers and thinking of ways to overcome them (BCT 5)—they had to read the questions multiple times. These questions were perceived as hard and annoying. Participants did not have any problems regarding the feedback that helped them solve their problems. One participant mentioned that the tailored feedback she received to overcome the barriers enabled her to plan more consciously.

Five participants agreed with the feedback on performance (BCT 6) and accepted it. One participant commented that it was objective, not too strict or pedantic, and initiated thinking about behavior, while 2 other participants found it too strict. One of them said she would not take the feedback into consideration. Participants were happy to receive compliments.

**Table 3:** Major usability issues and resolutions per round of testing.

Round	Type of problem	Problem emerged	Resolution
1	Layout	Information on increasing the font size was overlooked	Information was made more apparent
	Content	The work/school questions in the SQUASH were annoying and answered incorrectly	The answer option: “I do not have a job/I do not go to school” was added to skip the remaining job/school questions
		The assessment of intention and stages of change was perceived as unnecessary and because of that frustrating	A short explanatory introduction was added
		Program and feedback messages too lengthy	[no quick solution]
	Navigation	Scrolling after increased font size	[no quick solution]
			
2	Layout	Participant clicked in the wrong check box, when filling out answer options	An empty line was inserted after each 3 lines
	Navigation	Participants noticed the option to change font size, but did not succeed changing it	Instruction was simplified
	Content	The work/school questions in the SQUASH were annoying and answered incorrectly [solution not sufficient]	1. A warning to fill out the answer option:” I do not have a job/ I do not go to school” was added 2. A short overview of the questions was added 3. The sequence of the questionnaire was changed
		The assessment of intention and stages of change was perceived as unnecessary [solution not sufficient]	One question was removed
		Some feedback on physical activity disturbed the participant suffering from severe COPD	[no quick solution]
			
3	Navigation	One participant had problems changing the font size [solution not sufficient]	[no quick solution]

## Discussion

The purpose of this study was to assess and improve the usability of a computer-tailored program aimed at supporting self-management of COPD patients. We found a need for various improvements concerning layout, navigation, and most importantly content. This is in line with other studies evaluating the usability of eHealth applications [34,35]. A remarkable finding was from a participants’ comment that some feedback was not adequate for severe COPD patients because of their progressive physical limitations. This finding demonstrated that feedback should be tailored to the severity of disease.

This study had several limitations. One limitation was inherent to its pragmatic approach. While as few as 5 test subjects are considered enough to find the majority of usability problems [22] and the best revenues from usability evaluations come from iterative testing [23], such a limited number of participants (we used 8) is not enough to generate valid and reliable metrics that can be analyzed [36]. On the other hand, this pragmatic approach enabled us to correct most problems and retest the program with corrections implemented.

Another limitation was that we were unable to solve all usability problems that were found, given the time frame of the usability study and the technical means. For example, the problem associated with the lengthiness of the program remains unsolved. This becomes an issue particularly for future users of the program, who will not be aided by a moderator. Their frustration with the long program length may lead to discontinued use of the program. This is the reason why we decided for a post-hoc analysis of the BCTs to explore the possibility of making the program more elective rather than compulsory.

The question related to that issue is whether the selection of BCTs should be based on the stage of change [31], the users’ preferences [16], or other considerations. There are many studies that evaluate the impact of BCTs provided by computer-tailored programs. For example, a program can enhance the self-efficacy of carrying out certain behaviors of a user by assessing the individual’s perceived barriers to physical activity and giving feedback to increase confidence for dealing with the identified barriers. Another technique may be to invite persons to make action plans on how to prepare a new behavior [14,37,38]. Some BCTs used in eHealth interventions have been shown to be more effective than others [39]. However, less is known about how users appreciate the BCTs used in these programs [40]. BCTs have a different content in each study, and it is unclear which aspects appeal to the users.

The evaluation of responses in our study pertaining to the content of questions and feedback messages, based on the BCTs, showed that it is important to achieve an appropriate balance between positive and objective feedback considering performance (BCT 6). The rationale for this BCT is to stimulate behavior change [27]. However, the feedback needs to be carefully tested. Feedback that is too strict results in frustration while feedback that is not strict enough does not make patients aware of their unhealthy behavior. The comments on other BCTs varied. Some participants found the content useful, while others expressed a negative opinion. This is in line with the trans-theoretical construct that states that individuals move through stages of change and need a corresponding approach for each stage [31]. A number of computer-tailored programs provide individuals with the BCTs that match their stage of change [40]. However, the results of our study imply that users’ characteristics should also be taken into account when selecting BCTs. For example, BCTs that described consequences of various behaviors were not appreciated when the participant felt that he/she already had sufficient knowledge on the topic. Some participants had problems understanding or appreciating BCT 4, goal setting (behavior) and BCT 5, identifying barriers and thinking of ways to overcome these barriers, while others had no problems and appreciated these BCTs. A computer-tailored intervention that incorporates these BCTs could be more helpful for users who prefer to use intensive cognitive processes, such as active planning and problem solving. According to dual process models (eg, Petty and Cacioppo’s Elaboration Likelihood Model [41]), information can be processed via two routes, the central and the peripheral. The central route requires more cognitive effort and leads to more elaborations, whereas information processing via the peripheral route is more automatic. A personal preference for information processing, which can be measured by the need for cognition scale [42] or the information-processing questionnaire [43], could influence the processing intensiveness of BCTs 4 and 5. Nonetheless, simplifying the questions and feedback messages in BCT 4 and 5 and lowering the required effort to process the information could make these BCTs accessible for a broader population.

Several participants were reluctant to embrace BCTs that incorporated normative social influence. While the social environment can be an important factor to influence an individual’s behavior [44], some participants resisted acknowledging social influence on their behavior or their influence on others, as this would compromise their integrity or that of others. Deutsch et al [45] argued that normative social influence could undermine individual integrity. Hence, we recommend that BCTs containing normative social influence should be carefully applied to prevent users from feeling that their individual integrity could be compromised, but emphasize that individuals have independent judgments and learn from each other to accomplish behavior change.

This usability study was conducted to improve a computer-tailored program aimed at improving the self-management of COPD patients, but the usability methods followed in this usability study can be applied as part of a user-centered design in any eHealth interventions. This study uncovered several inconveniencies in the program that could be resolved, which shows that a usability evaluation with end users of an eHealth intervention is highly recommended. This study also revealed that the users appreciated BCTs implemented in this intervention. These results may be helpful for developers to consider which BCTs they should use in their eHealth interventions. Further research is needed to uncover which user characteristics affect the use of computer-tailored programs and the choice of BCTs that fit best.

## Abbreviations

BCT: behavior-change techniques
COPD: chronic obstructive pulmonary disease
MARS-5: Medication Adherence Report Scale
SQUASH: Short Questionnaire to Assess Health-Enhancing Physical Activity

## References

[1] Mathers CD, Loncar D (2006). Projections of global mortality and burden of disease from 2002 to 2030. PLoS Med.

[2] Viegi G, Pistelli F, Sherrill DL, Maio S, Baldacci S, Carrozzi L (2007). Definition, epidemiology and natural history of COPD. Eur Respir J.

[3] Bourbeau J, Julien M, Maltais F, Rouleau M, Beaupré A, Bégin R, Renzi P, Nault D, Borycki E, Schwartzman K, Singh R, Collet JP, Chronic Obstructive Pulmonary Disease axis of the Respiratory Network Fonds de la Recherche en Santé du Québec (2003). Reduction of hospital utilization in patients with chronic obstructive pulmonary disease: a disease-specific self-management intervention. Arch Intern Med.

[4] Effing T, Monninkhof EM, van der Valk PD, van der Palen J, van Herwaarden CL, Partidge MR, Walters EH, Zielhuis GA (2007). Self-management education for patients with chronic obstructive pulmonary disease. Cochrane Database Syst Rev.

[5] Bourbeau J, Nault D, Dang-Tan T (2004). Self-management and behaviour modification in COPD. Patient Educ Couns.

[6] Gaikwad R, Warren J (2009). The role of home-based information and communications technology interventions in chronic disease management: a systematic literature review. Health Informatics J.

[7] Bartoli L, Zanaboni P, Masella C, Ursini N (2009). Systematic review of telemedicine services for patients affected by chronic obstructive pulmonary disease (COPD). Telemed J E Health.

[8] Evers KE (2006). eHealth promotion: the use of the Internet for health promotion. Am J Health Promot.

[9] Krebs P, Prochaska JO, Rossi JS (2010). A meta-analysis of computer-tailored interventions for health behavior change. Prev Med.

[10] Noar SM, Benac CN, Harris MS (2007). Does tailoring matter? Meta-analytic review of tailored print health behavior change interventions. Psychol Bull.

[11] Strecher VJ, Shiffman S, West R (2005). Randomized controlled trial of a web-based computer-tailored smoking cessation program as a supplement to nicotine patch therapy. Addiction.

[12] Te Poel F, Bolman C, Reubsaet A, de Vries H (2009). Efficacy of a single computer-tailored e-mail for smoking cessation: results after 6 months. Health Educ Res.

[13] Prochaska JJ, Spring B, Nigg CR (2008). Multiple health behavior change research: an introduction and overview. Prev Med.

[14] de Vries H, Kremers SP, Smeets T, Brug J, Eijmael K (2008). The effectiveness of tailored feedback and action plans in an intervention addressing multiple health behaviors. Am J Health Promot.

[15] de Vries H, Brug J (1999). Computer-tailored interventions motivating people to adopt health promoting behaviours: introduction to a new approach. Patient Educ Couns.

[16] Hawkins RP, Kreuter M, Resnicow K, Fishbein M, Dijkstra A (2008). Understanding tailoring in communicating about health. Health Educ Res.

[17] Elfeddali I, Bolman C, de Vries H (2012). SQ4U - a computer tailored smoking relapse prevention program incorporating planning strategy assignments and multiple feedback time points after the quit-attempt: development and design protocol. Contemp Clin Trials.

[18] Eysenbach G (2005). The law of attrition. J Med Internet Res.

[19] Ahern DK (2007). Challenges and opportunities of eHealth research. Am J Prev Med.

[20] Wichansky AM (2000). Usability testing in 2000 and beyond. Ergonomics.

[21] Bevan N (1995). Usability is quality of use.

[22] Nielsen J, Landauer TK (1993). A mathematical model of the finding of usability problems.

[23] Nielsen J (1993). Iterative user-interface design. Computer.

[24] de Vries H, Mesters I, van de Steeg H, Honing C (2005). The general public's information needs and perceptions regarding hereditary cancer: an application of the Integrated Change Model. Patient Educ Couns.

[25] Smeets T, Kremers SP, Brug J, de Vries H (2007). Effects of tailored feedback on multiple health behaviors. Ann Behav Med.

[26] Abraham C, Michie S (2008). A taxonomy of behavior change techniques used in interventions. Health Psychol.

[27] Michie S, Ashford S, Sniehotta FF, Dombrowski SU, Bishop A, French DP (2011). A refined taxonomy of behaviour change techniques to help people change their physical activity and healthy eating behaviours: the CALO-RE taxonomy. Psychol Health.

[28] Heatherton TF, Kozlowski LT, Frecker RC, Fagerström KO (1991). The Fagerström Test for Nicotine Dependence: a revision of the Fagerström Tolerance Questionnaire. Br J Addict.

[29] Horne R, Hankins M, Jenkins R (2001). The Satisfaction with Information about Medicines Scale (SIMS): a new measurement tool for audit and research. Qual Health Care.

[30] Wendel-Vos GC, Schuit AJ, Saris WH, Kromhout D (2003). Reproducibility and relative validity of the short questionnaire to assess health-enhancing physical activity. J Clin Epidemiol.

[31] Prochaska JO, DiClemente CC, Norcross JC (1992). In search of how people change. Applications to addictive behaviors. Am Psychol.

[32] Jaspers MW, Steen T, van den Bos C, Geenen M (2004). The think aloud method: a guide to user interface design. Int J Med Inform.

[33] Hsieh HF, Shannon SE (2005). Three approaches to qualitative content analysis. Qual Health Res.

[34] Choi J, Bakken S (2010). Web-based education for low-literate parents in Neonatal Intensive Care Unit: development of a website and heuristic evaluation and usability testing. Int J Med Inform.

[35] Stinson J, McGrath P, Hodnett E, Feldman B, Duffy C, Huber A, Tucker L, Hetherington R, Tse S, Spiegel L, Campillo S, Gill N, White M (2010). Usability testing of an online self-management program for adolescents with juvenile idiopathic arthritis. J Med Internet Res.

[36] Dicks RS (2002). Mis-usability: on the uses and misuses of usability testing.

[37] van Stralen MM, de Vries H, Mudde AN, Bolman C, Lechner L (2009). Efficacy of two tailored interventions promoting physical activity in older adults. Am J Prev Med.

[38] van Keulen HM, Mesters I, Ausems M, van Breukelen G, Campbell M, Resnicow K, Brug J, de Vries H (2011). Tailored print communication and telephone motivational interviewing are equally successful in improving multiple lifestyle behaviors in a randomized controlled trial. Ann Behav Med.

[39] Webb TL, Joseph J, Yardley L, Michie S (2010). Using the internet to promote health behavior change: a systematic review and meta-analysis of the impact of theoretical basis, use of behavior change techniques, and mode of delivery on efficacy. J Med Internet Res.

[40] Lustria ML, Cortese J, Noar SM, Glueckauf RL (2009). Computer-tailored health interventions delivered over the Web: review and analysis of key components. Patient Educ Couns.

[41] Petty RE, Cacioppo JT (1986). The elaboration likelihood model of persuasion. Adv Exp Soc Psychol.

[42] Cacioppo JT, Petty RE, Kao CF (1984). The efficient assessment of need for cognition. J Pers Assess.

[43] Smerecnik CM, Mesters I, Candel MJ, De Vries H, De Vries NK (2012). Risk perception and information processing: the development and validation of a questionnaire to assess self-reported information processing. Risk Anal.

[44] Ajzen I (1991). The theory of planned behavior. Organ Behav Hum Decis Process.

[45] Deutsch M, Gerard HB (1955). A study of normative and informational social influences upon individual judgement. J Abnorm Psychol.

